# Mendelian Randomization in Case Only Studies: A Promising Approach to be Applied With Caution

**DOI:** 10.1016/j.amjcard.2018.09.035

**Published:** 2018-12-15

**Authors:** Ruth E. Mitchell, Lavinia Paternoster, George Davey Smith

**Affiliations:** MRC Intergrative Epidemiology Unit, University of Bristol, United Kingdom

In response to a recent study by Zafrir et al^1^ reporting an inverse association between excess adiposity and mortality rate in patients that underwent cardiac catherization, Trischitta and Di Paolo^2^ provide an insightful comment on the importance of addressing the “obesity paradox.” This study^1^ as well as others^3^, ^4^ show that increased adiposity appears seemingly protective against all-cause mortality^4^ as well as in patients with high-risk conditions including coronary artery disease,^1^, ^5^ type-II diabetes^6^ and end-stage renal disease.^7^ Trischitta and Di Paolo suggest that this paradoxical effect in frail and high-risk patients could be due to adipocytokine profile improving drugs or the presence of a preexisting disease that causes weight loss, and therefore the result of survival bias. Other factors may also results in bias such as confounding by age, ill-health and lifestyle factors as well as selection bias.^8^, ^9^, ^10^

To overcome these limitations, Trischitta and Di Paolo emphasize the need to investigate the causal relation of adiposity and/or body mass index (BMI) on mortality rate in high-risk patients directly through randomized controlled trials or using genetic variants in an instrumental variable approach through Mendelian randomization (MR).^2^ MR is a useful approach in investigating causal relations in the absences of confounding and reverse causality^11^ and as they point out, recent MR studies have supported the causal role of higher BMI on higher risk of coronary heart disease using publicly available GWAS data^12^ and (more recently) on increasing the risk of all-cause mortality and specifically cardiovascular disease in UK Biobank.^13^

**Figure 1:**
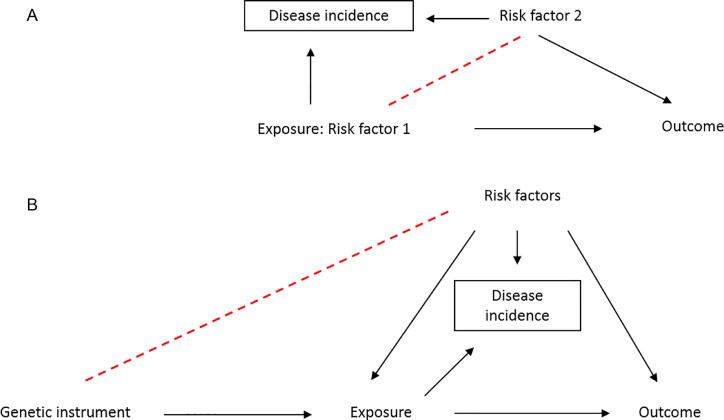
DAG demonstrating the introduction of collider bias in case-only studies. (A) Risk factors, both genetic and nongenetic, become spuriously associated due to risk factors being independently associated with disease incidence (*dashed line*). This opens up a noncausal pathway from exposure to outcome when a risk factor is also associated with the outcome. (B) In MR analysis, the genetic instruments cause the disease and therefore they become spuriously associated with independent risk factors which are confounders of the exposure-outcome. Conditioning on incidence opens noncausal pathways between all variables that cause incidence. DAG = direct acyclic graph; MR = Mendelian randomization.

We agree with Trischetta and Di Paolo, that epidemiological studies within selected groups of individuals (e.g., cases of a particular disease, or individuals at known increased risk) is a promising approach to identify causal relations. However, it must also be taken into account that proposing to apply epidemiological methodologies (including MR) in “well-powered samples of frail patients”^2^ will also be subject to potential collider bias by design, due to studying a selected group of individuals, as we have recently described.^14^ Indeed, disease incidence becomes a collider variable due to its association with other independent risk factors for being a case of that disease (genetic and nongenetic), resulting in those risk factors becoming spuriously associated in the cases. When the risk factors are also associated with the outcome, conditioning on this collider (i.e., selecting case only individuals) opens up a noncausal path (Figure 1).^14^ In the case of a genetic study within cases, this will result in spurious associations between genetic variants and the outcome.^15^ Therefore, increased care needs to be taken to address potential confounding in studies of nonrandom group of individuals.

Specifically, in the context of MR studies, the assumption that the genetic instrument is independent of factors that confound the associations of the exposure and the outcome may be violated.^14^ If the exposure causes the onset of disease, then the genetic instruments for the exposure may become associated with other independent risk factors for onset in a group of cases.^14^ In other words, a spurious association is induced between the gene and confounders of the exposure-outcome association leading to a noncausal association between the gene and the outcome (Figure 1).^16^ When investigating obesity as the exposure and mortality rate as the outcome in high-risk patients, selecting individuals based on having coronary heart disease for example, may induce spurious associations between BMI (and therefore, genetic variants related to BMI) and confounders such as age, educational attainment, smoking, and alcohol intake.

This collider bias can lead to biased and misleading estimates of causal associations^17^ and to an over- or under-identification of risk factors for the outcome of interest in a nonrandom sample.^14^ Therefore, it is important to assess the presence, magnitude, and direction of the bias, which will depend on the exact nature of the combined effects of variables on disease status and the relation between variables.^14^ We suggest a number of ways to detect and correct for this bias, including inverse probability weighting when the effects of confounders on disease onset are known.^14^ This is an area of current ongoing methodological research and should be taken into consideration not just in MR studies but in any studies of case only samples.

Trischitta and Di Paolo finish by highlighting that results from MR studies can inform the selection of targets for randomized controlled trials.^2^ This is a very promising approach^18^ and as pointed out “MR studies conducted in well-powered samples of frail patients are timely needed.”^2^ These types of studies offer considerable opportunity to identify treatment targets and inform therapeutics. Therefore, large-scale sources of data with measures of progression along with genetic data need to be sought out and studies designed to appropriately address collider bias.
